# A global database of food and nutrient consumption

**DOI:** 10.2471/BLT.15.156323

**Published:** 2016-09-16

**Authors:** Shahab Khatibzadeh, Michael Saheb Kashaf, Renata Micha, Saman Fahimi, Peilin Shi, Ibrahim Elmadfa, Shadi Kalantarian, Pattra Wirojratana, Majid Ezzati, John Powles, Dariush Mozaffarian, on behalf of the Global Burden of Diseases Nutrition, Chronic Diseases Expert Group (NutriCode)

**Affiliations:** a Harvard School of Public Health, Boston, United States of America (USA).; b Tufts Friedman School of Nutrition Science & Policy, 150 Harrison Avenue, Boston, MA 02111, USA.; c Institute of Nutritional Sciences, University of Vienna, Vienna, Austria.; d School of Public Health, Imperial College London, London, England.; e School of Clinical Medicine, University of Cambridge, Cambridge, England.

In every region of the world, poor diet is a leading cause of both malnutrition and chronic diseases including diabetes, cardiovascular diseases and specific cancers.^1^^–^^3^ In 2013, 38.3 million deaths occurred due to chronic diseases globally (70% of all deaths), with most of these deaths occurring in developing countries.^4^ Anecdotal evidence and more formal evaluations in a limited number of countries suggest that changes in traditional eating patterns and a growing reliance on new types of foods are major drivers of these transitions. However, data on global patterns of dietary habits, as well as differences by population characteristics are not well established. An empirical assessment of dietary intakes is needed for evidence-based policy-making to address global health challenges.

In most nations worldwide, assessment of dietary habits has been limited by the absence of robust data on individual dietary intakes that can be used in comparative studies. Up to now, most global analyses have evaluated only single dietary factors or have used data on crude household expenditure or national food supply estimates that do not adequately capture individuals’ actual consumption levels.^5^^,^^6^ Moreover, types of foods consumed and diet-related diseases are often unevenly distributed within populations and it is therefore essential to collect data on specific demographic groups to understand the impact of diets on diseases. Furthermore, even when individual dietary intakes are available, these are rarely standardized or comparable across countries or time, due to differences in the data collection instruments and their intended use, in the design and administration of surveys, and in data processing and analysis.

As part of our efforts for the 2010 Global Burden of Diseases study, we systematically identified the available data from national and subnational surveys of individual-based dietary intakes of key foods and nutrients worldwide, by age, sex, country and time (1980–2010). Our preliminary methods have been reported^7^ and further details are available from the corresponding author on request. Briefly, we searched multiple electronic databases and used extensive personal communications with researchers and government authorities worldwide to identify and obtain nationally representative dietary intake surveys or, if these were unavailable, large subnational surveys. For countries without identified national or subnational individual-level dietary surveys, we searched for individual-level surveys from large cohort studies as well as other data sources on diet such as the World Health Organization (WHO) Global Infobase, the WHO STEPS database and household expenditure surveys. For trans-unsaturated fatty acids (trans-fats) and dietary sodium, we also searched for biomarker surveys measuring circulating or adipose trans-fat concentrations or 24-hour urinary sodium excretion. Finally, we used the comprehensive United Nations Food and Agricultural Organization (FAO) food balance sheets,^8^ which provide country-level data on per capita food availability for major food groups in 187 countries and across the entire time period studied. For trans-fat, we also included industry estimates of nation-specific availability of partially hydrogenated oil, total oils/fats and total packaged foods per capita from both retail and food-service establishments in 79 countries (Mark Stavro, Bunge LLC, personal communication, 23 May 2012). Due to the limited amount of relevant published data, most survey data were obtained by direct contacts with researchers and officials.

By combining all these sources of information, including adjusted FAO data and industry estimates, our final estimates were derived from dietary information drawn from 187 countries. We included data from 325 dietary surveys and 145 urinary sample surveys. The total number of individuals sampled in each surveyed country ranged from several hundred to more than 10 000. The dietary surveys were from 116 countries representing around 3923 million adults: 88.7% of the global adult population of 4422 million in 2010. The urine sample surveys were from 52 countries representing 3 181 million adults: 71.9% of the global adult population (Table 1).

**Table 1:** General characteristics of the data included in the Global Dietary Database

Variable	Individual-level dietary surveys	Individual-level 24-hour urine surveys	FAO food balance sheets^8^
Total no. of surveys	325	145	187^a^
Total no. of individuals in the surveys	1 747 236	54 448	NA
No. of countries represented	116	52	187
Global adult population represented in 2010, millions^b^	3 923	3 181	4 422
**Year of collection,^c^ no. (%) of surveys**			
1980–1997	151 (46.5)	109 (75.2)	187 (NA)^d^
1998–2010	174 (53.5)	36 (24.8)	187 (NA)^d^
**Geographical representativeness, no. (%) of surveys**			
National	233 (71.7)	13 (9.0)	187 (100.0)
Regional	63 (19.4)	97 (66.9)	0 (0.0)
Urban, rural, or other subnational cohort	29 (8.9)	35 (24.1)	0 (0.0)
**Dietary assessment method,^e^ no. (%) of surveys**			
Multiple (2+) diet recalls or records	63 (19.4)	NA	NA
Food frequency questionnaire	89 (27.4)	NA	NA
Single short-term diet recalls or records	99 (30.5)	NA	NA
Simple food survey or household expenditure survey	78 (24.0)	NA	NA
24-hour urine collection	NA	145 (100.0)	NA
National food availability	NA	NA	187 (100.0)
**Sample size, no. (%) of surveys**			
< 1000	94 (28.9)	134 (92.4)	NA
1000–5000	133 (40.9)	11 (7.6)	NA
5001–10 000	30 (9.2)	0 (0.0)	NA
> 10 000	68 (20.9)	0 (0.0)	NA
**Data source,^f^ no. (%) of surveys**			
Published papers or reports	98 (30.2)	140 (96.6)	0 (0.0)
Data provided by corresponding members^g^	124 (38.2)	5 (3.4)	0 (0.0)
Individual-level data from public sources or provided by corresponding members^g^	53 (16.3)	0 (0.0)	187 (100.0)
DAFNE database	54 (16.6)	0 (0.0)	0 (0.0)

DAFNE: Data Food Networking; FAO: United Nations Food and Agriculture Organization; NA: not applicable.

^a^ Total number of countries included in this analysis, with separate annual estimates for each country over the years 1980–2010. The following United Nations (UN) Member States were not included in the FAO database: Andorra, Liechtenstein, the Marshall Islands, Monaco, Palau, Timor-Leste and Tuvalu. Cook Islands is not a UN Member State, but is included in the FAO database.

^b^ The total population of UN countries excluded from the FAO database is 1 489 180. This is likely an overestimate of the population of these countries at the time of the analysis.

^c^ Or first year of survey, if multiple years.

^d^ FAO food balance sheets provide entry-level data on per capita food availability for major food groups in 187 countries and across the entire time period studied.

^e^ The total exceeds 325 as some surveys included more than one dietary assessment method.

^f^ The total exceeds 325 as data for some surveys were retrieved from more than one source. Further details of the data sources are available from the corresponding author.

^g^ Due to the limited amount of relevant published data, most survey data were obtained by direct contacts with researchers and officials.

Note: The data sources were combined to create a global database of dietary intakes. We standardized survey measurements by accounting for within- versus between-person variation to assess distributions of intakes, assessing differences in categorizations of dietary factors and their measurement units, and adjusting for total energy intake. A Bayesian hierarchical model incorporated differences between surveys and FAO data. The model included individual-level survey data and statistical uncertainty by age, sex, country and time; differences in geographical representativeness, categorizations of food groups and dietary assessment methods; FAO data, including up to 17 foods/nutrients and four factors derived from principal components analysis; industry data (for trans-fats); country’s gross domestic product; and random effects by country, 21 world regions and seven world super-regions. We gave greater statistical weight in the model to national versus subnational surveys, primary versus secondary categorizations of foods/nutrients, and individual versus household dietary surveys. Model validity was evaluated by cross-validation.

We assessed the distributions of consumption within each country by age, sex and time period, using standardized methods across countries and surveys. To account for expected heterogeneity in the surveys, we used systematic extraction and analysis methods while also evaluating and incorporating differences in survey characteristics and geographical representativeness into our final dietary estimates. The definitions of dietary metrics and their units were standardized across surveys and selected to correspond to those used in previous research to assess the evidence of disease–diet relationships. Dietary intakes were adjusted for total energy intake to reduce measurement error and also account for differences in activity, body size and metabolism; a second analysis without this adjustment derived similar results. A hierarchical estimation model accounted for the size and statistical certainty of each survey, differences in survey versus FAO data (which often overestimate true intakes) and heterogeneity in geographical representativeness and comparability of surveys (and the consequent effects on statistical uncertainty).

The resulting Global Dietary Database covers 21 key foods and nutrients identified as relevant to risk of chronic diseases:^7^ total energy, fruit, 100% fruit juice, vegetables, beans/legumes, nuts/seeds, whole grains, red meats, processed meats, seafood, milk, sugar-sweetened beverages, saturated fat, omega-6 polyunsaturated fat, seafood-derived omega-3 fat, plant-derived omega-3 fat, trans-fat, dietary cholesterol, dietary fibre, dietary (and urinary) sodium, and dietary calcium (see Table 2 for details on global coverage and definitions of each; available at: http://www.who.int/bulletin/volumes/94/12/15-156323).

**Table 2:** Available data on consumption of foods and nutrients used to generate the Global Dietary Database

Dietary **factor**	Individual or household surveys^a^							Available dietary variables (optimal definition^d^)
	No. of surveys	No. of surveys with individual-level assessment (%)	No. of surveys with age- and sex- specific data (%)	Year range^b^	No. of countries covered	% of global adult population covered^c^	World region covered	
Total energy	120	110 (91.7)	98 (81.7)	1980–2010	66	79.2	AE, APH, AS, ASE, AUS, CAR, EURC, EURE, EURW, LAC, LAS, LAT, NA, NAM, OC, SSE, SSS, SSW	Total energy
Fruit	214	147 (68.7)	123 (57.5)	1980–2010	109	85.2	AC, AE, APH, AS, ASE, AUS, CAR, EURC, EURE, EURW, LAC, LAS, LAT, NA, NAM, OC, SSC, SSE, SSS, SSW	Total fruits, (including fresh, frozen, cooked, canned or dried fruit; excluding fruit juices and salted or pickled fruit)
100% fruit juice^d^	127	64 (50.4)	58 (45.7)	1980–2010	46	48.9	AE, APH, ASE, CAR, EURC, EURE, EURW, LAT, NA, NAM, SSS	Total fruit juice (100% juice)
Vegetables	214	147 (68.7)	123 (57.5)	1980–2010	109	85.2	AC, AE, APH, AS, ASE, AUS, CAR, EURC, EURE, EURW, LAC, LAS, LAT, NA, NAM, SSC, SSE, SSS, SSW	Total vegetables (including fresh, frozen, cooked, canned or dried vegetables; excluding salted or pickled vegetables, vegetable juices, starchy vegetables [e.g. potatoes, corn], legumes, nuts and seeds)
Beans/legumes	148	82 (55.4)	72 (48.7)	1980–2010	64	81.2	AE, APH, AS, ASE, AUS, CAR, EURC, EURE, EURW, LAC, LAS, LAT, NA, NAM, SSE, SSS, SSW	Total beans and legumes (including tofu; excluding soy milk)
Nuts/seeds	136	71 (52.2)	64 (47.1)	1980–2010	53	73.6	AE, APH, AS, ASE, AUS, CAR, EURC, EURE, EURW, LAC, LAS, LAT, NA, NAM, SSE, SSS, SSW	Total nuts and seeds (can include peanuts, peanut butter)
Whole grains	39	39 (100.0)	39 (100.0)	1987–2010	25	40.9	AE, APH, AS, ASE, AUS, CAR, EURC, EURE, EURW, LAC, LAS, LAT, NA, NAM, SSE, SSS, SSW	Total whole grains (including whole grain breakfast cereals, bread, rice, pasta, biscuits, muffins, tortillas, pancakes)
Red meats, unprocessed	164	97 (59.1)	79 (48.2)	1980–2010	74	82.7	AC, AE, APH, AS, ASE, AUS, CAR, EURC, EURE, EURW, LAC, LAS, LAT, NA, NAM, SSE, SSS, SSW	Total red meat (including beef, pork, lamb, both domesticated and game; excluding poultry, fish, eggs all processed meats; may include offal)
Processed meats	137	70 (51.1)	68 (49.6)	1980–2010	54	53.6	AE, APH, ASE, AUS, CAR, EURC, EURE, EURW, LAC, LAS, LAT, NA, NAM, SSS	Total processed meat (including processed deli or luncheon meats [ham, turkey, chicken, pastrami, etc.], bacon, salami, sausages, bratwursts, frankfurters, hot dogs)
Seafood	125	58 (46.4)	50 (40.0)	1980–2010	52	53.7	AE, APH, AS, ASE, AUS, CAR, EURC, EURE, EURW, LAC, LAT, NA, NAM, SSE, SSS, SSW	Total seafood (including fish and shellfish)
Milk	167	102 (61.1)	79 (47.3)	1980–2010	75	82.6	AC, AE, APH, AS, ASE, AUS, CAR, EURC, EURE, EURW, LAC, LAS, LAT, NA, NAM, SSE, SSS, SSW	Total milk (including non-fat, low-fat, and full-fat milk; excluding soya milk or other plant-derived alternatives)
Sugar sweetened beverages	127	73 (57.5)	65 (51.2)	1980–2010	52	50.6	AE, APH, ASE, AUS, CAR, EURC, EURE, EURW, LAC, LAS, LAT, NA, NAM, SSS	Total sugar sweetened beverages: (including any beverage with added sugar and ≥ 50 kcal per 8 oz [226.8 g], such as carbonated beverages, soft drinks, sodas, energy drinks, fruit drinks, etc.; excluding 100% fruit and vegetable juices)
Saturated fat	85	85 (100.0)	81 (95.3)	1980–2010	49	70.3	AE, APH, AS, ASE, AUS, CAR, EURC, EURE, EURW, LAC, LAT, NA, NAM, OC, SSS	Total saturated fat (from all dietary sources, primarily meat, dairy products, and tropical oils)^d^
Omega-6 polyunsaturated fat	61	61 (100.0)	61 (100.0)	1986–2010	33	46.6	AE, APH, ASE, AUS, CAR, EURC, EURE, EURW, LAC, LAT, NA, NAM, SSS	Total omega-6 polyunsaturated fat (from all dietary sources, primarily liquid vegetable oils such as soya bean, corn and safflower)^d^
Omega-3 polyunsaturated fat, seafood-derived	116	62 (53.4)	54 (46.6)	1980–2010	57	59.0	AE, APH, AS, ASE, AUS, CAR, EURC, EURE, EURW, LAC, LAT, NA, NAM, SSE, SSS, SSW	Total dietary eicosapentaenoic and docosahexaenoic acid (from all dietary sources, primarily seafood; excluding supplements)
Omega-3 polyunsaturated fat, plant-derived	32	32 (100.0)	32 (100.0)	1990–2010	21	44.1	AE, APH, CAR, EURC, EURW, LAC, LAT, NA, NAM, SSS	Total dietary α-linolenic acid (from all dietary sources; excluding supplements)
Trans-unsaturated fatty acids	60	50 (83.3)	25 (41.7)	1980–2010	23	18.8	APH, AS, CAR, EURW, LAC, LAT, NA, NAM, SSS	Total trans-unsaturated fat (from all dietary sources, mainly partially hydrogenated vegetable oils and ruminant products)
Dietary cholesterol	80	80 (100.0)	75 (93.8)	1980–2010	46	53.0	AE, APH, AS, ASE, AUS, CAR, EURC, EURE, EURW, LAC, LAS, LAT, NA, NAM, OC, SSS	Total dietary cholesterol (from all dietary sources)
Dietary fibre	87	87 (100.0)	77 (88.5)	1980–2010	53	71.2	AE, APH, AS, ASE, AUS, CAR, EURC, EURE, EURW, LAC, LAS, LAT, NA, NAM, OC, SSS	Total dietary fibre (from all dietary sources, primarily fruits, vegetables, grains, legumes, pulses; excluding supplements)
Sodium (dietary surveys)	117	116 (99.1)	113 (96.6)	1986–2010	46	66.4	AE, APH, AS, ASE, AUS, CAR, EURC, EURE, EURW, LAC, LAS, LAT, NA, NAM, OC, SSS	Total dietary sodium (from all dietary sources)
Sodium (urinary surveys)	145	145 (100.0)	145 (100.0)	1990–2009	52	71.9	APH, AC, AE, AS, ASE, AUS, CAR, EURC, EURE, EURW, LAC, LAS, LAT, NAM, NA, OC, SSE, SSS, and SSW	Total excreted sodium over 24 hours
Dietary calcium	100	100 (100.0)	88 (88.0)	1980–2010	60	74.9	AE, APH, AS, ASE, AUS, CAR, EURC, EURE, EURW, LAC, LAS, LAT, NA, NAM, OC, SSE, SSS, SSW	Total dietary calcium (from all dietary sources; excluding supplements)

AC: Asia, central; AE: Asia, eastern; APH: Asia–Pacific, high income; AS: Asia, southern; ASE: Asia, south-eastern; AUS: Australasia, CAR: Caribbean; EURC: Europe, central; EURE: Europe, eastern; EURW: Europe, western; LAA: Latin America, Andean; LAC: Latin America, central; LAS: Latin America, southern; LAT: Latin America, tropical; NA: North America, high income; NAM: North Africa/Middle East; OC: Oceania; SSC: sub-Saharan Africa, central; SSE: sub-Saharan Africa, eastern; SSS: sub-Saharan Africa, southern; SSW: sub-Saharan Africa, western.

^a^ In addition to these surveys, for all countries we included data from the comprehensive United Nations Food and Agricultural Organization (FAO) food balance sheets, which provide country-level data on per capita food availability for major food groups in all 187 countries and across the entire time period studied (1980–2010). The FAO data were matched by major food sources or transformed for certain nutrients (e.g. for omega-6 polyunsaturated fat, using the major seed oils, weighted by their percentage content of omega-6 polyunsaturated fat; and for dietary sodium, using four factors from principal components analysis of 17 major FAO good groups). Data on trans-unsaturated fatty acids were supplemented with industry estimates of nation-specific availability of partially hydrogenated oil, total oils/fats and total packaged foods per capita from both retail and food-service establishments in 79 countries. These FAO and industry data were used in a hierarchical Bayesian model to estimate consumption of the primary dietary metric of interest, based on the relationship between this variable and our data from individual-level surveys among countries having data on both.

^b^ Or first year of survey, if multiple years.

^c^ Based on approximate global adult population of 4 422 million in 2010.^9^

^d^ For each food category, we requested and obtained data from each survey corresponding to the specific definitions listed here. When data based on the optimal definition were not available, we obtained data based on the most similar available definition and accounted for these differences in our Bayesian hierarchical model to derive final global estimates.

Data sources: Further details of the data sources are available from the corresponding author.

We believe that the database provides the best available estimates of the mean (and standard deviation) intakes of key dietary factors by age, sex, country, region and time period. The categorization of dietary factors is designed to correspond as closely as possible with the definitions used in prospective studies and controlled trials that have quantified the harmful or protective effects of diet on noncommunicable diseases.^7^ Before this effort, no comprehensive global database existed on the intakes of these foods and nutrients that each have public health relevance. FAO food balance sheets provide important information on average national food availability, but not on actual intakes or on heterogeneity within populations.^10^ The WHO Global InfoBase assesses only fruit and vegetable consumption in mostly developing countries.^11^ The European Nutrition and Health Report^11^ and Data Food Networking^12^ databases offer robust intake and household expenditure or consumption data, but this is limited to Europe. To build on and leverage existing work, each of these data sets was incorporated into our effort. The Global Dietary Database collates the best available evidence on global dietary intakes, and further standardizes and unites these data through quality assessment and quantitative modelling (Table 3).

**Table 3:** Comparison of global and regional dietary databases and variables incorporated into the Global Dietary Database

Variable	Global Dietary Database	Source database			
		WHO Global InfoBase^11^	European Nutrition and Health Report^11^	Data Food Networking database^12^	FAO food balance sheets^8^	
No. of dietary factors assessed	21	3	20	15	101 commodities	
Age-specific estimates available	Yes	Yes	No	No	No	
Sex-specific estimates available	Yes	Yes	No	No	No	
No. of world regions covered^a^	21	15	3	3	21	
No. of countries covered	187	94	25	24	187	
% of the global adult population covered^b^	98.6	43.1	8.9	8.9	98.6	
No. of surveys incorporated	411^c^	121	2 rounds	70	6 rounds	
Urinary sodium assessed	Yes (24-hour collection surveys)	No	No	No	No	
Years included	1980–2010	2001–2013^d,e^	2004, 2009	1981–2004^e,d^^,f^	1961–2013^e^^,f^	
Geographical representativeness of surveys	National level for 86.5% of dietary surveys and 23.9% of 24-hour urine surveys	Mixed	National	National	National	
Dietary assessment tools	Bayesian modelling, including diet records and recalls, FFQ, household budget surveys, FAO food balance sheets industry data to estimate trans-fat, other covariates, and statistical uncertainty	FFQ	National food availability estimates, FFQ, household surveys, diet records or recalls	Household budget surveys	National food supply estimates (food balance)	

FAO: United Nations Food and Agriculture Organization; FFQ: food frequency questionnaires; trans-fat: trans-unsaturated fatty acids; WHO: World Health Organization.

^a^ Based on the 21 Global Burden of Disease world regions.^2^

^b^ Based on approximate global adult population of 4422 million in 2010.^9^

^c^ Including both dietary surveys and 24-hour urine surveys. The Global Dietary Database further incorporated each of the additional data sources in this table, as well as, for trans-fat, industry estimates of nation-specific availability of partially hydrogenated oil, total oils/fats and total packaged foods per capita from both retail and food-service establishments.

^d^ Data range varies across individual countries.

^e^ Fewer countries are included in the earlier years.

^f^ Survey year varies across the studied countries; the range of years is provided.

We did not assess diets in childhood, by urban versus rural location or by socioeconomic status. Ongoing work should address these gaps by 2018.^13^ Separate, individual-level national surveys were not available for every country, dietary factor and time period; this meant that we needed to increase the statistical uncertainty and reliance on modelling and adjusted FAO data in these cases. The surveys varied in their national representativeness, age groupings, dietary instruments and dietary categorizations; we minimized these effects by using standardized survey assessment, data retrieval methods, analysis methods and hierarchical modelling.

These data have broad implications for public health research and policy. The Global Dietary Database has been made available to researchers and can be requested online (http://www.globaldietarydatabase.org/). Systematic global data on dietary intakes are important for quantifying the disease burden attributable to suboptimal diets.^2^ Assessing diets by age, sex and time is important for understanding differences within populations and analysing trends over time. The database will allow scientists, governments and transnational organizations to identify intervention targets for nutrition programmes and initiatives to reduce the burden of diet-related diseases.

The data also offer an assessment of the scope of global dietary surveillance. Fruits and vegetables were the most frequently assessed dietary factor in individual-level surveys, included in 214 surveys from 109 countries (Table 2). Plant-derived omega-3 fatty acids were the most rarely assessed (32 surveys), although these data came from 21 populous nations comprising 1950 million people, nearly half (44.1%) of the global adult population. The lowest population coverage from individual-level surveys was for trans-fats: 60 surveys from 23 countries, representing 831 million people (18.8% of the world’s adult population); these data were therefore supplemented with industry estimates from 79 countries as described above. Patterns in data availability identify key gaps in surveillance in developing nations, particularly in sub-Saharan Africa, and these can inform efforts to expand dietary surveillance. For example, among world regions, sub-Saharan Africa had the fewest available individual-level dietary data, and mostly only on fruits and vegetables from the WHO Global InfoBase.

In conclusion, we combined systematic survey searches with extensive personal contacts to derive a global database of dietary habits. The Global Dietary Database addresses several key limitations of prior data sources, combining broad global coverage with estimates of food and nutrient consumption by age, sex and time. We believe that these data provide an empirical basis for global dietary surveillance, policy-making and priority setting to address diet-related burdens of disease.

## References

[1] Danaei G, Ding EL, Mozaffarian D, Taylor B, Rehm J, Murray CJ, et al. The preventable causes of death in the United States: comparative risk assessment of dietary, lifestyle, and metabolic risk factors. PLoS Med. 2009 4 28;6(4):e1000058. 10.1371/journal.pmed.1000058 19399161 PMC2667673

[2] Lim SS, Vos T, Flaxman AD, Danaei G, Shibuya K, Adair-Rohani H, et al. A comparative risk assessment of burden of disease and injury attributable to 67 risk factors and risk factor clusters in 21 regions, 1990-2010: a systematic analysis for the Global Burden of Disease Study 2010. Lancet. 2012 12 15;380(9859):2224–60. 10.1016/S0140-6736(12)61766-8 23245609 PMC4156511

[3] Lozano R, Naghavi M, Foreman K, Lim S, Shibuya K, Aboyans V, et al. Global and regional mortality from 235 causes of death for 20 age groups in 1990 and 2010: a systematic analysis for the Global Burden of Disease Study 2010. Lancet. 2012 12 15;380(9859):2095–128. 10.1016/S0140-6736(12)61728-0 23245604 PMC10790329

[4] GBD 2013 Mortality and Causes of Death Collaborators. Global, regional, and national age-sex specific all-cause and cause-specific mortality for 240 causes of death, 1990-2013: a systematic analysis for the Global Burden of Disease Study 2013. Lancet. 2015 1 10;385(9963):117–71. 10.1016/S0140-6736(14)61682-2 25530442 PMC4340604

[5] Claro RM, Jaime PC, Lock K, Fisberg RM, Monteiro CA. Discrepancies among ecological, household, and individual data on fruits and vegetables consumption in Brazil. Cad Saude Publica. 2010 11;26(11):2168–76. 10.1590/S0102-311X2010001100018 21180990

[6] Naska A, Berg MA, Cuadrado C, Freisling H, Gedrich K, Gregoric M, et al.; Data Food Networking (DAFNE) participants. Food balance sheet and household budget survey dietary data and mortality patterns in Europe. Br J Nutr. 2009 7;102(1):166–71. 10.1017/S000711450809466X 18986595

[7] Micha R, Kalantarian S, Wirojratana P, Byers T, Danaei G, Elmadfa I, et al.; Global Burden of Diseases, Nutrition and Chronic Disease Expert Group. Estimating the global and regional burden of suboptimal nutrition on chronic disease: methods and inputs to the analysis. Eur J Clin Nutr. 2012 1;66(1):119–29. 10.1038/ejcn.2011.147 21915137

[8] FAOSTAT [Internet]. Rome: Statistics Division, Food and Agriculture Organization of the United Nations; 2015. Available from: http://faostat3.fao.org/home/E [cited 2016 Jul 22].

[9] World population prospects, the 2015 revision. New York: Population Division, United Nations; 2015. Available from: https://esa.un.org/unpd/wpp/ [cited 2016 Jul 22].

[10] Kelly A, Becker W, Helsing E. Food balance sheets. Food and health data: their use in nutrition policy-making. Copenhagen: WHO Regional Office for Europe; 1991 pp. 39–47. 1750979

[11] Ng N, Van Minh H, Tesfaye F, Bonita R, Byass P, Stenlund H, et al. Combining risk factors and demographic surveillance: potentials of WHO STEPS and INDEPTH methodologies for assessing epidemiological transition. Scand J Public Health. 2006;34(2):199–208. 10.1080/14034940500204506 16581713

[12] Lagiou P, Trichopoulou A; DAFNE contributors. DAta Food NEtworking. The DAFNE initiative: the methodology for assessing dietary patterns across Europe using household budget survey data. Public Health Nutr. 2001 10;4(5B) 5b:1135–41. 11924937

[13] Global Nutrition and Policy Consortium. Home of the Global Dietary Database [Internet]. Boston: Tufts Friedman School of Nutrition Science and Policy; 2016. Available from: http://www.globaldietarydatabase.org [cited 2016 Jul 7].

